# Developing a Framework for Culturally Sensitive Breastfeeding Interventions: A Community Needs Assessment of Breastfeeding Experiences and Practices in a Black Immigrant Community

**DOI:** 10.3390/nu17132094

**Published:** 2025-06-24

**Authors:** Temitope Awelewa, Alexandra Murra, William T. Story

**Affiliations:** 1 Stead Family Department of Pediatrics, Carver College of Medicine, University of Iowa, Iowa City, IA 52242, USA; 2 Carver College of Medicine, University of Iowa, Iowa City, IA 52242, USA; alex-murra@uiowa.edu; 3 Department of Community and Behavioral Health, College of Public Health, University of Iowa, Iowa City, IA 52242, USA; william-story@uiowa.edu

**Keywords:** breastfeeding, breast milk expression, culture, barriers, immigrant

## Abstract

Background/Objectives: Despite high breastfeeding initiation rates nationwide, disparities in breastfeeding continuation among Black mothers remain a public health issue. The BreastFeed Iowa Black Immigrant Project aimed to improve breastfeeding rates among low-income Black immigrant mother-infant dyads in Johnson County, Iowa by exploring factors influencing breastfeeding retention and developing a framework for culturally sensitive breastfeeding interventions. Methods: Using a descriptive cross-sectional study design, we employed a convergent, parallel mixed-methods approach to explore factors that influence breastfeeding duration among Black immigrant mothers with children 0–2 years old. Nine focus group discussions (FGDs) were conducted on Zoom among 40 participants with a semi-structured guide on breastfeeding beliefs, experiences, and feeding practices. Additionally, a 22-item survey was administered to 33 participants. Results: Based on the survey, one out of five participants indicated that they were unable to breastfeed for as long as they had planned. The top five reasons why mothers stopped breastfeeding included having received formula from the Supplemental Nutrition Program for Women, Infants and Children; being sick and having to take medicine; the baby was hungry too often; the baby was sick and could not breastfeed; and not producing enough milk. The top five themes that emerged from FGDs as barriers to breastfeeding included lack of lactation support, knowledge gaps on breastfeeding benefits, perception of inadequate milk supply, lack of comfort with breast milk expression, and work-related barriers. Conclusions: The community needs assessment identified the elements needed to develop a culturally sensitive framework with targeted interventions to address breastfeeding barriers in the Black immigrant community.

## 1. Introduction

The health benefits of breastfeeding as the standard feeding recommendation for an infant are well documented [1,2,3]. The American Academy of Pediatrics recommends exclusive breastfeeding for 6 months, with the addition of complementary food at 6 months and breastfeeding continuation for 2 years and beyond as desired. Breastfeeding benefits are higher with longer breastfeeding duration for both the mother and the child [1,3]. Breastfeeding initiation rates are high, but retention rates remain a nationwide problem [4]. About 8 out of 10 infants initiate breastfeeding; however, only half of all infants are still breastfeeding at 6 months [5,6,7].

Breastfeeding initiation and retention rates are consistently lower among communities that experience marginalization [8,9]. Breastfeeding retention at 6 months is lowest among non-Hispanic Blacks, a group that includes African immigrants, with a 14 percentage point difference compared to White populations for infants born in 2019 [4,6]. Disparities in national breastfeeding trends among non-Hispanic Blacks may mirror trends in breastfeeding practices among Black immigrant mothers [8]. Many factors have been correlated with breastfeeding trends across different ethnic groups [10]. Low-income mothers attending the Special Supplemental Nutrition Program for Women, Infants and Children (WIC) are less likely to breastfeed compared to non-eligible mothers [11,12]. Lower breastfeeding retention rates in general have been associated with sociocultural barriers such as lack of perceived social support, the need to return to work, and lack of a breastfeeding-friendly job [13]. Black women are more likely to experience socioeconomic barriers that mitigate breastfeeding [12]. Moreover, Black immigrant mothers from diverse backgrounds and of low socio-economic status experience significant challenges seeking culturally sensitive care upon immigration to high-income countries [14,15].

Despite low breastfeeding retention rates among Black mothers, there is little information on the breastfeeding challenges experienced by diverse African immigrant communities that contribute to the national breastfeeding disparities in the United States [5,16]. There is also a paucity of breastfeeding intervention frameworks that are specifically designed based on a breastfeeding needs assessment among low-income Black immigrant communities [17]. The BreastFeed Iowa Black Immigrant Project aims to improve breastfeeding retention rates among low-income Black immigrant mother-infant dyads in Johnson County, Iowa by conducting a community needs assessment. Johnson County is the fourth most populous county in Iowa [18], and it has the highest proportion of foreign-born residents compared to neighboring Iowa counties [19]. In 2022, 41% of Iowa refugees were from the Democratic Republic of Congo, and about 6% were from Eritrea [20]. Iowa’s immigrant and refugee population is growing rapidly, with a 23% increase between 2020 and 2023 [21]. Further, 8.8% of births in Iowa occur to people who do not identify English as their primary language [22]. Therefore, in Iowa’s predominantly rural, White context, there is a need for creative community-driven responses to improve the health and well-being of the changing population.

This study aims to (1) explore breastfeeding beliefs and factors influencing breastfeeding retention among Black immigrants in Johnson County, and (2) develop a framework for culturally sensitive breastfeeding interventions among Black immigrants. The findings from the community needs assessment presented in this paper will inform the design of a targeted breastfeeding quality improvement project that addresses the unique socio-cultural needs of Black immigrants in Johnson County, Iowa by improving access to culturally sensitive lactation support services. There are currently no projects in the Johnson County area, or in the State of Iowa, focused on improving breastfeeding rates in the Black immigrant community.

## 2. Materials and Methods

This study employed a descriptive cross-sectional study design using a community needs assessment. Specifically, this observational study employed a convergent, parallel, mixed-methods approach with focus groups and interviewer-assisted surveys to explore factors influencing breastfeeding duration among low-income Black immigrant mothers with children 0–2 years in Johnson County, Iowa, USA. Low income level was assumed based on WIC eligibility [23]. This study used mixed methods, which is in line with a recent analysis of research studies on breastfeeding among Black women that showed most studies utilize quantitative research methods, demonstrating a need for more studies to include qualitative methods for a broader grasp of breastfeeding experiences in Black women [17].

The community needs assessment emphasized community engagement in all phases—from the design of the study to the dissemination of the results. Community partnership involved quarterly principal investigator (PI) meetings with the local breastfeeding coalition and representatives from the WIC office and the Congolese Health Partnership over the project period from 2021 to 2023. Partners provided input on planned project activities, grant applications, and the creation of a community advisory group that assisted with recruiting participants for the community needs assessment. Community advisory group members included two mothers with prior mixed breastfeeding experience and one with no prior breastfeeding experience.

The three community advisory group members assisted with the recruitment of participants—Black immigrant women who ever breastfed with a child of 0–2 years in the community. Recruitment flyers were developed to include information about the project, eligibility, and information for participating in the study. The flyers were widely distributed among community partners, in local clinics, shopping centers, community centers, and in apartment buildings. Interested community participants self-enrolled into the study by contacting community advisory members who provided more information on study participation in participant-preferred languages.

The University of Iowa IRB (IRB ID# 202106401) determined that informed consent could be waived because this was a quality improvement project aimed at improving breastfeeding rates in the community. The information shared by participants was without identifiers and was not linked to individuals. Moreover, participants were informed to only answer the questions with which they felt comfortable. Participants were screened for eligibility by community advisory board members and the project coordinator before participating in the study.

The first component of this mixed-methods study was the focus group discussion (FGD), after which the participants were invited to participate in an online survey. Focus groups have been shown to be ideal for getting robust information from individuals in culturally and linguistically diverse settings [24]. The number of focus groups was determined by saturation of themes; focus group sessions were stopped when no added information was forthcoming from participants [25,26]. Nine FGDs were conducted online on Zoom among 40 participants (averaging 4 participants per focus group) from August to December of 2021. A semi-structured focus group guide (Table 1) was pre-tested by the project coordinator and PI with community advisory board members. Following the pre-test, the focus group guide was used by the PI to facilitate FGDs. The guide assessed participant breastfeeding knowledge, attitudes, feeding practices, breastfeeding barriers, experiences with lactation support, and suggestions for lactation interventions, with time for open discussion at the end of the sessions. All FGDs were translated in real time into French, Lingala, and Swahili by the three community advisory group members. Each session lasted between 60 and 105 min. Sessions were audio-recorded, transcribed in English, and analyzed for major themes.

Transcripts were reviewed by both the project coordinator and PI for common themes. Data were analyzed using thematic analysis to examine views and opinions about breastfeeding [27,28]. The transcripts were separately analyzed by the project coordinator and PI for key words and statements that were used to generate major themes. Thematic analysis allowed the team to uncover deeper meanings and significance in the transcripts. Using this strategy, an inductive coding approach was employed to generate a codebook categorizing participant responses under recurring themes. The final code structure was used to report concepts and findings, and, where relevant, verbatim quotations from the focus groups were included to illustrate key points or themes.

The three community advisory board members were also trained to administer a 22-item online survey to participants using Qualtrics. Thirty-seven participants screened as eligible for the online survey, and 33 participants completed the survey. Most (*n* = 26) of the participants were from the Democratic Republic of Congo. Other countries included Togo (*n* = 2) and Sudan (*n* = 1) while four nationalities were unknown. The surveys were translated into the three most common languages spoken in the community—French, Lingala, and Swahili. The survey question items aimed to identify participant sociodemographic characteristics; breastfeeding knowledge, attitudes, and practices; access to lactation services; breast pump perceptions; and workplace breastfeeding support. Participants received a $25 gift card as compensation for their time participating in the project. Quantitative data analysis involved calculating percentages and frequencies using Qualtrics. Due to the descriptive nature of this study, further statistical tests were not necessary.

## 3. Results

### 3.1. Online Survey

**Figure 1:**
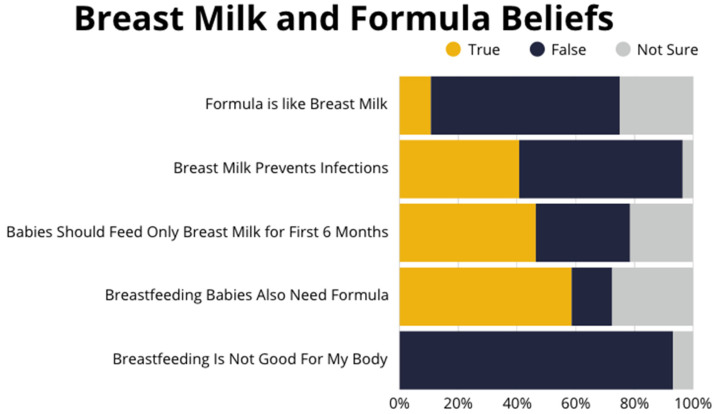
Beliefs about breast milk vs. formula-feeding—survey responses.

The social and demographic characteristics of the participants are displayed in Table 2. Most participants indicated that they intended to breastfeed for 1–2 years. However, about one in five participants said they were not able to breastfeed for as long as they wanted to. The average age participants stopped breastfeeding their children varied from 3 weeks to a maximum of 1 year. Over half of the participants indicated receiving formula from WIC as a reason for their decision to stop breastfeeding or to feed their baby formula (Table 3) and felt that breastfeeding babies also need formula (Figure 1). The majority (*n* = 29) of participants felt breastfeeding was good for their body; however, about one-third of participants were unaware of or unsure of the benefits of breast milk and felt that breast milk is like formula (Figure 1).

**Figure 2:**
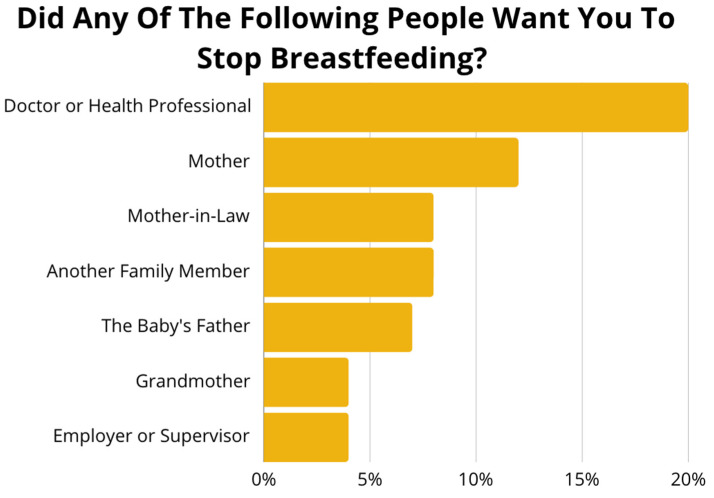
Survey responses to the question, “Did any of the following people want you to stop breastfeeding?” Frequencies indicate the percentage of participants who indicated “Yes”.

Regarding breastfeeding support, participants answered if they were asked to stop breastfeeding by any family members, employers, or health professionals. More participants indicated that a doctor or health care professional asked them to stop breastfeeding compared to other family members or employers (Figure 2). Participants were also asked how supportive their place of employment was. Over one-third of participants said their employer was either not supportive at all or not too supportive. About four of five participants said they received no assistance with breastfeeding their child, while only one out of ten said they received assistance from either their pediatrician or nutritionist. Regarding experiences with lactation support, one-third reported that they did not know how to get help, and about one out of five participants stated that their health insurance status prevented them from seeking help.

Participants were asked several questions about breast pump use and beliefs. About one-third of participants indicated that they did not own an electric breast pump. Twenty-four out of twenty-nine participants said they were uncomfortable with using a breast pump. No participants agreed to being able to figure out the use of the breast pump without being shown directions. About half of participants indicated that a lactation consultant, WIC staff, nurse, or doctor showed them how to use a breast pump. Only one-fifth of participants with a breast pump read the printed directions that came with the pump, and only two participants read or watched instructions on the internet.

### 3.2. Focus Group Results

#### 3.2.1. Summary of Major Topics

The major topics that emerged from the FGDs were breastfeeding knowledge, attitudes, and beliefs; infant feeding practices; breastfeeding support differences between their home country and the United States; perception of inadequate milk supply; work-related breastfeeding barriers; attitudes towards the use of breast pumps and donor milk; lactation support and culturally sensitive hospital care; and community education needs. The major themes were further organized by common themes around breastfeeding barriers.

Many participants reported that they did not feel comfortable feeding their babies breast milk left in the breast for long periods after work. Many women in the community stopped breastfeeding after returning to work. Many women expressed concern about insufficient education provided by providers on the benefits of breastfeeding and communication issues with the use of interpreters. Many expressed a desire for breastfeeding education and support in preferred spoken languages. Most discussed that breastfeeding barriers were work-related barriers, perceptions of not being able to produce enough milk, discomfort and unfamiliarity with using a breast pump, perceived safety of expressed breast milk, lack of education about the benefits of breastfeeding compared to formula-feeding, stigma with breastfeeding in public, fear of exposing the baby to the SARS-CoV-2 virus, and concern about breasts losing shape after breastfeeding.

Below are major topics discussed along with quotes from participants. Refer to Table A1 for additional quotes from participants.

#### 3.2.2. Breastfeeding Knowledge, Attitudes, and Beliefs

Many participants expressed beliefs about the benefits of breastfeeding, while some were unaware of breastfeeding benefits and the superiority of breastfeeding over formula-feeding. Some women expressed concern about breastfeeding in public and the long-term effect of breastfeeding on the appearance and shape of the breast.

“Breastfeeding in my culture is very important because we believe it makes the child stronger and smart.”

mother from Democratic Republic of Congo

“I do not see the difference between breast milk and formula. However, when I had my baby in Illinois, I was told about the difference between the two and encouraged to breastfeed. But for me, I don’t see any difference.”

mother from Democratic Republic of Congo

“For example, when you go to Walmart with your child and your child is crying and you need to give breast, but you can’t give your child your breast because people will be looking at you weirdly. Like what are you about to do? However, back home you can give your child the breast anywhere in public. And people don’t really care about you feeding your child. But the challenge comes here when you think, “Okay, I’m going to take this baby out, but starts crying I won’t be able to give it breast milk.” So, it’s just better to switch to formula as its comfortable and you don’t have to always worry about giving breast when you are out with the child.”

mother from Democratic Republic of Congo

#### 3.2.3. Infant Feeding Practices with Potential to Affect Breastfeeding

Many participants reported providing their newborns with water and complementary feeds for concerns about constipation. Many expressed the culture in their home country was to start solids and water as early as 2 months. Some participants were misinformed by other community members that the nutrition from breast milk was not good enough after they were directed to fortify breast milk with formula in the hospital.

“We, as older people, we drink water when we are hot because it makes us to feel good. And the kid too, sometimes they are hot, and they need water to cool down. So why do the doctor always say we should not be giving the kids water? I think it is not okay.”

mother from Democratic Republic of Congo

“I had a sister who had a baby here. After delivering the baby [she] got sick. So, she started using the pump because her breasts were filling up. After some time, they were mixing the milk in the freezer with formula. So, the reason they were giving them that [was because] the breast milk was losing some of its nutrition, so they needed to add some formula. So, after I left the hospital, I started doing that too, mixing the formula and the breast milk. I was wondering if that was the right thing to do.”

mother from Democratic Republic of Congo

#### 3.2.4. Breastfeeding Support Experiences in Home Country vs. the U.S.

Participants reported challenges with single parenting and getting family support with breastfeeding in the U.S. as opposed to their home country. Many expressed a desire for specific lactation-related nutrition education.

“In Africa we got lots of support from family members and we got a whole lot of time to rest. We had a lot of time to breastfeed our child. But here, based on our occupations and work schedule, we don’t get enough time to breastfeed our child as long as possible.”

mother from Democratic Republic of Congo

“So, especially for single mothers. You live by yourself; you take care of all kids by yourself; you don’t have time to go through all that or [have] someone to help you with all of that.”

mother from Democratic Republic of Congo

“Back home, we are exposed to so many things that produce milk. The food and stuff to do that is really easy to get. And so that we can have milk. However, here we are lost. We don’t know what to do, what to eat, or how to go about it in order to get milk.”

mother from Democratic Republic of Congo

#### 3.2.5. Perception About Inadequate Milk Supply

Many participants expressed concerns about inadequate milk supply and a desire to continue breastfeeding when the baby refused to breastfeed. Many expressed a desire for doctors to address inadequate milk supply and provide directions on how to increase supply when the baby refuses to breastfeed.

“My first baby, I breastfed for a year and 8 months, but for the second one I breastfed for a whole year. But this is the first time I am having a baby and every time I give the baby milk, the baby ends up crying without getting enough milk. Now I am wondering—I have tried everything. I have tried to eat vegetables only and things like that. But it is not working, I am still short in my milk. And I don’t understand the cause of that.”

mother from Democratic Republic of Congo

“One thing I would like the doctors to do to come up with something that helps us have milk after I have babies. I have noticed since I have come here, every time I have a baby that it is hard for my milk to come out for more than 5 months. I am not sure if it is related to what I am eating. But I would like, at least, for the doctors to tell us what to eat while breastfeeding so that we are able to breastfeed longer. And I have noticed that I am not the only person. I have met a lot of Africans who are having the same issue where the milk does not come out at all, or it doesn’t come out for long. So, if the doctor can at least tell us what to eat and what not to eat. Because I have asked the question before, and I was told that there is nothing—there is no medication to take in that situation. So now, I am wondering what to eat and what not to eat.”

mother from Democratic Republic of Congo

#### 3.2.6. Work-Related Breastfeeding Barriers

Participants mentioned work-related barriers including maternal fatigue after work, early return to work, formula-feeding while the mother is at work, lack of breastfeeding-friendly work environments, and engorgement and breast pain at work. Many mothers expressed beliefs about breast milk going bad during long hours of work and did not feel comfortable feeding their babies breast milk left in the breast for long periods after work because of concerns that it is not good milk. Many expressed that babies refuse breast milk after mothers return from a long day at work without breastfeeding or pumping.

“The difficulty was work. It was not easy because I had to return to work from early in the morning till late at night. During that time, the baby had to have formula. Or if I pumped and put it in the fridge, they could have that. But it wasn’t easy.”

mother from Togo

“Sometimes it is very hard. We are working and the breast gets very big and it’s hurting but we have to keep working like that for 10 h. When we come home, the breast is coming up with so much pressure such that when we give it to the baby the baby starts coughing and they won’t want to take the breast anymore.”

mother from Democratic Republic of Congo

“I am afraid of giving my baby my breast milk because it has been in there for so long after work, it goes bad.”

mother from Democratic Republic of Congo

#### 3.2.7. Attitude Towards Use of Breast Pump

Only a few participants felt that pumping was beneficial and achievable. Many reported concerns about pain when pumping. Many did not pump at work and reported that their work environment was not conducive to pumping and breast milk storage. There were also concerns about the safety of pumping, with fear of potential unknown health consequences to the mother and the potential for infection for the child due to contamination of breast milk.

“I don’t pump at work because I don’t think that’s a good place to do it. I’d have to go do it in the restroom and I don’t think that’s a clean place to do it. I also feel like I don’t have enough time to go pump, so I prefer to do it at home.”

mother from Democratic Republic of Congo

“I am sure there is a difference from when the baby is breastfeeding and when I am pumping. I feel like pumping is pulling the breast too hard. And now I am kind of worried that the way it does there will be consequences in the future… Is it not going to cause any breast cancer?”

mother from Democratic Republic of Congo

“Pumping at work is a challenge. Because my workplace has a room where we can go and pump, but we don’t have a fridge to put it in until we finish work.”

mother from Democratic Republic of Congo

#### 3.2.8. Attitude Towards Breast Milk Donation and Use of Donor Milk

Some participants felt that donating breast milk would be acceptable in certain situations when a baby needs it and has no other choice, like when a mother is deceased. However, many participants felt maternal breast milk is personalized for the baby, were against the use of donor breast milk for their babies, and would rather give their babies formula. A participant expressed concern about transmission of infection through donor milk.

“Donation is not bad; it is good to give milk for other babies. Because there are some parents who give birth and then they die. But for me, I am alive, so I don’t see myself putting my baby in that situation. Or taking donor milk. It is best for me to just keep trying to give breast milk and if I can’t then I give formula.”

mother from Democratic Republic of Congo

“I cannot take somebody else’s breast milk. Instead, I’d take the other option where I wait for the breast milk to come out. I might take some water mixed with sugar and give it to the child until I have breast milk.”

mother from Democratic Republic of Congo

#### 3.2.9. Lactation Support and Culturally Sensitive Hospital Care

Many participants were concerned that health care professionals did not provide or spend enough time on breastfeeding education. Many expressed a desire for more culturally sensitive education during the antenatal period and immediate postpartum hospital stay. Some stopped breastfeeding because of misinformation from health care professionals on the compatibility of illness and medications used with lactation.

“I think that preparing someone for breast milk should start when they are pregnant. And, since I have been going to the doctor here when pregnant, I never seen the doctor or nurse discussing breastfeeding.”

mother from Democratic Republic of Congo

“When I went to the hospital the doctors ask me if I am going to breastfeed or not. But they did not educate me on why it is important to give breast milk. And I think that when they start doing that more women will be more likely to breastfeed rather than just jumping to formula.”

mother from Democratic Republic of Congo

“For my case it was because I had high blood pressure, and the doctor wanted to prescribe a medication and asked me to stop breastfeeding so that I would be able to take the medicine.”

mother from Democratic Republic of Congo

#### 3.2.10. Tailored Community Education Needs

Many participants expressed a need for breastfeeding education and a desire for communication in preferred non-English languages. Many prefer audiovisuals to written information and recommend peer-to-peer information sharing by community members for education needs. Group educational sessions and community sessions with leaders were identified as important forums to connect with the community.

“The doctor might have given them education regarding breastfeeding. But I think the problem is with the translator. Because some translators have an accent or speak a different French than what they do. For example, they might have a Canadian accent, and we are from Congo and a lot of us speak with a Belgian accent, so it is hard for us to understand.”

mother from Democratic Republic of Congo

“Many people do not have the culture of reading just because of being busy with kids and other stuff. But if there’s a video then people can just play it in the background.”

mother from Democratic Republic of Congo

“Back home there are groups for women who just had kids… I also think that it shouldn’t be like WIC where they look at your income and you might be ineligible to attend. It should be somewhere where it is free for everyone to come. Like, I didn’t know that I could pump milk and it’s good to stay for up to 4 h. So, I’m just hearing it right now. And I know there are women like me who have never heard about it. That it would be really educational to bring all of them together in one room and try to educate them.”

mother from Democratic Republic of Congo

## 4. Discussion

This descriptive, cross-sectional study was based on a community needs assessment that identified factors that influence breastfeeding practices in a Black immigrant community and the need for access to culturally sensitive breastfeeding education and lactation support services. Our FGD and survey findings showed that barriers to breastfeeding included a lack of knowledge about the benefits of breastfeeding, lack of culturally sensitive lactation support, perception of inadequate milk supply, work-related barriers, and lack of comfort with breast milk expression.

Breastfeeding initiation rates are high nationwide, but very few women are able to breastfeed exclusively for 6 months [4] and for as long as desired. Odom et al. reported that six out of ten mothers stopped breastfeeding earlier than intended [13]. One in five participants in our community assessment did not breastfeed as long as intended. A nationally representative sample of breastfeeding mothers in the U.S. showed significant differences in breastfeeding continuation among subgroups of breastfeeding mothers based on country of birth. Non-Hispanic Black (NHB) mothers had lower rates of breastfeeding continuation when compared to a similar cohort of Hispanic mothers born in the U.S. Similarly, Caribbean-born Hispanic mothers had lower odds of any breastfeeding when compared to South and Central America-born Hispanic mothers and non-Hispanic White (NHW) mothers [9]. In a separate longitudinal study of diverse women enrolled in the WIC program, NHB and Hispanic women were less likely to meet breastfeeding intentions at 3 months compared to NHW women [29].

Participants in our community needs assessment indicated the need for increasing access to culturally sensitive lactation assistance and breastfeeding education. The perception about inadequate milk supply and that the baby was hungry were cited as top reasons for stopping breastfeeding. The American Academy of Pediatrics recommends frequent breastfeeding assessments and hands-on support in early postpartum care [3]. However, the majority (80%) of participants indicated that they received no assistance while breastfeeding. The top reasons for not receiving lactation assistance included not knowing how to get help and lack of insurance or the perception that insurance would not pay for services. Many participants expressed concern about inadequate breastfeeding education from providers. Similarly, about a third of participants in a small qualitative study of Black women in Greater New Haven reported lack of access to lactation support as a breastfeeding barrier [30]. Breastfeeding education and lactation support have been shown to reduce the risk of breastfeeding cessation at 6 months [31]. Safon et al. [9] found that access to breastfeeding information contributed to higher breastfeeding continuation among U.S. Hispanic mothers when compared to NHB mothers born in the U.S. Many participants expressed a desire for lactation support to address concerns about inadequate milk supply.

Knowledge gaps on the benefits of breastfeeding compared to formula-feeding exist among this group despite known benefits of breast milk feeding compared to formula-feeding [1]. About one-third of participants were unaware or unsure of how breast milk differs from formula. Receiving formula was the foremost reason for breastfeeding cessation. This is similarly reported in other studies among culturally diverse ethnicities [22,24]. A similar breastfeeding benefit knowledge gap was reported among Hispanic women in a qualitative study of WIC participants in Nevada. Hispanic women in the study perceived distribution of formula by the WIC program as an indication of WIC’s affirmation of formula use as an equally acceptable option to breastfeeding [32].

Health care professionals’ breastfeeding advice, or lack thereof, can limit breastfeeding knowledge and reduce breastfeeding continuation. Patients can be influenced by perceived negative breastfeeding attitudes from healthcare professionals [31]. Our survey findings showed that healthcare professionals were the most reported to want participants to stop breastfeeding. A national survey of healthcare professionals showed that many physicians have a negative attitude towards breastfeeding success, with only about six out of ten physicians stating that breastfeeding can be successful [33]. A review of breastfeeding literature across different settings similarly reported lack of practical information from health care providers as a barrier to breastfeeding continuation [34].

Work-related barriers prevent women from continuing to breastfeed. Workplace environments for low-income women appear to be less supportive of breastfeeding and breast milk expression [12]. Similar to studies in low- to middle-income countries, we found that work was a major barrier to breastfeeding retention among this group of low-income, immigrant women [35]. Many women in the FGDs highlighted the difficulty with breastfeeding after returning to work. About one-third of participants indicated that their employer was not supportive of breastfeeding. Work hours and work environments are reported to be non-conducive for breast milk expression. Similarly, a study of African American women in Detroit showed that return to work was a major barrier to breastfeeding continuation [36]. A study on the interaction of race and employment type in breastfeeding continuation showed that both White and Black women in service/labor jobs had lower breastfeeding duration compared to those in professional jobs. Snyder et al., however, reported that higher-income women employed within the professional and management industry were more likely to receive breastfeeding support upon return to work [37].

Many reported breast pain and engorgement at work, which, if persistent, spirals into unsuccessful breastfeeding maintenance. Other qualitative studies in culturally diverse settings have reported similar findings, including challenges with breast pain, exhaustion on return to work, and lack of workplace support as barriers to breastfeeding continuation [23,25]. Conversely, more than half of Black women in a study conducted among Black/African American women in Greater New Haven expressed that workplace policies had little to no effect on their breastfeeding decisions [30]. Mothers also expressed that babies refuse breast milk when mothers are engorged after work. Education on prevention of breast engorgement and breast milk expression on return to work can mitigate this barrier. Breastfeeding-friendly workplace policies can encourage mothers to express milk at work.

Breast milk expression is needed for continued maintenance of lactation for women who return to work and/or spend long hours away from the baby. However, many FGD participants expressed concern about the inferiority and safety of expressed breast milk. The majority (80%) of participants did not feel comfortable with using a breast pump at work and with storing expressed breast milk, and many expressed concerns about the safety of breast pump use. Although there are no known published studies on breast milk expression attitudes of low-income Black immigrant mothers returning to work in the U.S., a study among African women in Kenya demonstrated a knowledge gap on breast milk expression and storage [38]. Targeted education on breast milk expression can help low-income mothers in cross-cultural communities understand the need for breast milk expression and increase mothers’ self-confidence in pumping and safe breast milk storage.

A common topic that emerged from FGDs centered on cultural beliefs on the use of donor milk, and many expressed disapproval of the use of donor milk for their babies due to cultural beliefs and fear of disease transmission. A similar study of a small group of Ghanaian immigrant women in the U.S. showed that the women had mixed feelings and superstitious beliefs about the use of donor human milk [39]. The sentiments expressed in our FGDs are akin to studies conducted among breastfeeding mothers in African countries [40]. This suggests that many Black immigrant mothers may hold on to cultural beliefs from their country of origin. Cultural beliefs may prevent mothers from adopting the use of donor milk in settings where donor milk is recommended, like in the high-risk neonatal intensive care environment. Minority women may be unaware of the safety of donor human milk. Nine out of ten women who donated human milk to a cross-section of milk banks in the U.S. were educated NHWs with a bachelor’s or graduate degree [41]. A literature review of barriers to use of donor human milk also reported perceived safety concerns as a deterrent to receiving donor milk [42]. Targeted education on storage and safety of expressed breast milk and safe use of donor milk may help mothers increase appropriate use of expressed maternal milk and donor human milk.

It is well known that early introduction of complementary feeds is not nutritionally beneficial and can also negatively influence breastfeeding duration [43,44,45]. Many participants reported introducing complementary feeds as early as 2 months, which could have interfered with breastfeeding. Differing infant feeding beliefs, attitudes, and practices have been reported among Black children. An analysis of a national survey of children’s health in 2022 showed that 19.5% of Black children were introduced to complementary foods before 4 months as compared to 6.2% of White children [46]. Similarly, WIC participants in a small qualitative study of Hispanic women in Nevada practiced early introduction of supplemental foods [32].

African immigrants have varied educational backgrounds and acculturation experiences that may hinder adoption of infant feeding recommendations. Recognition of the unique cultural challenges and differences is invaluable in developing targeted education in diverse communities [14].

### 4.1. Programmatic Implications and Future Directions

**Figure 3:**
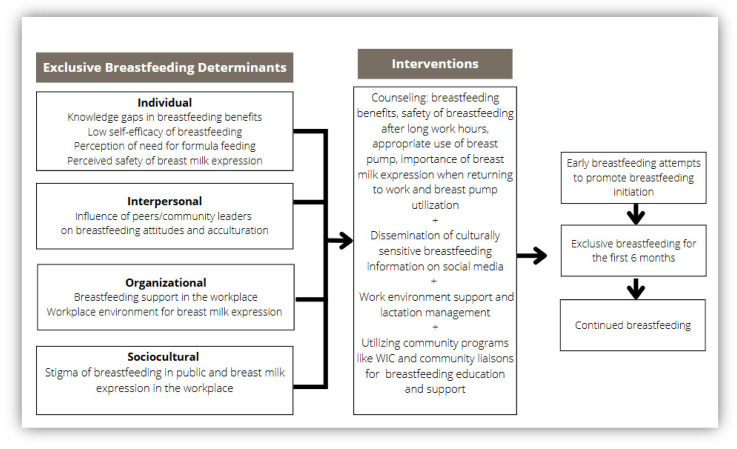
Determinants of exclusive breastfeeding in a Black immigrant community: A culturally sensitive breastfeeding intervention framework.

We used the themes from the FGDs to develop a framework [28] for breastfeeding interventions that address breastfeeding determinants at various levels of the socio-ecological model [47] (Figure 3). The interventions suggested in Figure 3 address the knowledge gap on breastfeeding benefits, safety of expressed breast milk, using culturally sensitive audiovisuals for disseminating breastfeeding education, accessing lactation support at work, and leveraging the influence of community leaders/peers for education and support. According to this new framework, breastfeeding retention is influenced by individual attributes such as a mother’s understanding of the safety and benefits of breast milk, confidence in her ability to adequately feed the baby with breast milk alone, and the impact of acculturation on formula-feeding decisions. Further, perceptions of breast pump safety and utilization influence the practice of breast milk expression after returning to work. Interpersonal factors include influences from peers, family members, and community members regarding breastfeeding. Finally, the framework includes larger organizational and sociocultural factors that affect access to lactation services and support, as well as social norms related to breastfeeding. Public health advocates should consider multi-level constructs to provide targeted breastfeeding interventions and promote policies that support breastfeeding in the workplace, reducing stigma of public breastfeeding and use of donor breast milk, and fear of breast milk expression in workplaces.

As a result of this study, community-focused interventions were developed to improve health care professionals’ engagement in culturally sensitive breastfeeding education and support. Interventions were developed that focused on addressing gaps in knowledge of breastfeeding benefits, accessing lactation services, use of breast pumps, leveraging community programs, and the influence of interpersonal relationships on breastfeeding attitudes. In line with the identified knowledge gap, the team developed breastfeeding education materials distributed in five non-English languages. Educational materials included topics on benefits of breastfeeding, resolving commonly encountered breastfeeding problems, breast pump utilization in the workplace, infant feeding practices, safety, and storage of breast milk.

The community needs assessment results are being used to develop a breastfeeding cultural guide for lactation consultants. This guide will inform the development of a culturally sensitive breastfeeding support program and empower lactation professionals (peer counselors, nurses, lactation educators, and consultants) with information needed to provide culturally sensitive care in the community. Responses from the FGDs demonstrate a need for tailored education materials translated with visual guides. Future implementation will include the development of interactive breastfeeding education videos.

Lactation support programs and policies are needed to support breastfeeding in every workplace environment, especially for low-income women whose jobs may not provide a supportive environment [48]. Public health efforts are needed to reduce the stigma of breastfeeding in public and fear of breast milk expression in workplace environments of low-income women. Health care professionals, public health advocates, and lactation professionals need to actively demonstrate support for breastfeeding by providing targeted breastfeeding education and interventions among low-income Black women [49].

### 4.2. Strengths and Limitations

The strength of this community needs assessment included the use of mixed methodology to get wide-ranging feedback from community members. We employed community advisory members as liaisons to increase community participation with appropriate language services to engage participants. Limitations of this project included a small sample size; however, focus group sessions were discontinued after we achieved saturation of themes as earlier discussed. Another limitation was that the focus group sessions were conducted at the tail end of the COVID-19 pandemic, from August to December of 2021 online. Participants were asked to reflect on past and current breastfeeding experiences; however, the difficulties with breastfeeding expressed by participants might have been influenced by recency bias with difficulties navigating the pandemic. This project was completed as a quality improvement project focused on improving breastfeeding in an immigrant community. Eligible participants were screened but self-enrolled into the community needs assessment. Findings may not be generalizable to other settings. The study needs to be replicated in different immigrant communities.

## 5. Conclusions

African immigrants have varied educational backgrounds and diverse acculturation experiences, necessitating a multi-contextual approach to curating targeted and culturally appropriate interventions. Major barriers to breastfeeding retention in different communities include communication barriers, scarce breastfeeding information from providers, lack of supportive workplace environments, and concerns about health insurance. Culturally sensitive lactation support, specific education on breastfeeding benefits, and lactation maintenance are needed to help with promoting breast milk expression among low-income Black immigrant mothers returning to work. This project highlights the importance of concerted breastfeeding support across different settings in the socioecological model.

## Figures and Tables

**Table 1:** Questions from the Focus Group Guide.

What do you think is the ideal feeding method for an infant? Probe further: What are the benefits of breastfeeding compared to formula-feeding? What influenced your decision to breastfeed or formula feed?
How would you describe your breastfeeding experiences when you were in your home country compared to your experiences since you immigrated? What is the prevailing attitude towards infant feeding in your home country? Do you have any specific newborn feeding practices in your home country?
What are your current practices with feeding your child? What are your beliefs about introducing solid food and water?
What are your personal experiences with breastfeeding? Were you able to breastfeed?
What are your experiences with using the breast pump? Ask further: Do you have prior experiences using the breast pump? Do you feel comfortable expressing breast milk with a breast pump? Did you feel supported to express breast milk at your workplace?
What are your perceptions on the use of donor milk for a newborn child?
What are your experiences with lactation support after you had your baby? Did you feel supported with your lactation support needs after you had your baby? Are you aware of how to get lactation assistance?
What are your experiences with the care you received in the hospital after you had your baby? What suggestions do you have for culturally sensitive care?
Open discussion. Any suggestions on how to connect community members with resources? Do you need assistance with accessing health care resources?

**Table 2:** Online survey demographics.

Survey Participant Demographics (n = 33)	
Mean age	34
Mean number of children aged 0–2 years	3
Highest level of education	College: 48% High school: 34% High school or no formal education: 16%
Mean number of years lived in the U.S	5.7 years
Married	97%
Insurance Status	Had health insurance: 76% (*n* = 25) Did not have health insurance: 12% (*n* = 4) Did not respond: 12% (*n* = 4)

**Table 3:** Top five reasons why mothers stopped breastfeeding.

Reason	% of Participants Who Indicated That This was a Very Important Reason for Breastfeeding Cessation
(1) Receiving formula/milk from WIC	52.2
(2) Mother was sick and had to take medicine	22.7
(3) Baby was hungry a lot of the time	21.7
(4) Baby was sick and could not breastfeed	18.2
(5) Not having enough milk production	16.7

## Data Availability

The original contributions from the FGDs presented in this study are included in the article. Further inquiries on the raw data supporting the conclusions of this article can be directed to the corresponding author.

## Abbreviations

The following abbreviations are used in this manuscript.

WIC	Women, Infants and Children
FGD	Focus group discussions

## Appendix A. Focus Group Transcript Details

**Table A1:** Other focus group discussion (FGD) quotes.

**Breastfeeding knowledge, attitudes, and beliefs** “Breast milk gives affection between mother and child.” “Breast milk can heal a baby.” “Breastfeeding provides emotional connection with the child which is unachievable with pumping.” “And when you put your child close to your chest you can feel if they have a fever, that connection. That stuff can be felt from there.” “[I] also realized after talking to my neighbors who are newer mothers. They stopped breastfeeding their child because they wanted to keep their body better looking.” “Most African women nowadays … don’t want to breastfeed because they don’t want their breast to like “fall off.” So, they want to keep it a certain look.”
**Infant feeding practices with potential to impact breastfeeding** “In my country, most of the time when the child is around 2 to 3 months that’s when I start giving them something like soup. So, it is solid food, but not milk. Something that will start to introduce the child to solid food. And around 5 months that’s when you can give them fufu which is a casava bread.” “I give my baby water because I think that breast milk has sugar, and it stays in the baby’s throat. So, I need to give the baby water to clear it out.” “One of the reasons why I have been giving water early [is] because the milk here I noticed that it causes a digestion issue and constipation. And when I give water it helps the child poop.” “In Congo, …… we start giving the baby food at 3 months. At that point they start mixing breast milk with things like solid food.”
**Breastfeeding support experiences in home country vs. the United States** “The difference is that here, most women work. So, they only have to breastfeed for 3 months. Compared to home, a lot of women do not work so they do breastfeed longer.” “Something warm like soup with veggies or tea helps the mother increase her breast milk for her to breastfeed the child later on. So, giving something warm to the mother before helps the mother have a lot of milk.”
**Perception about inadequate milk supply** “I don’t have milk.” “Since back home, I have never had milk.” “When I start to breastfeed, milk stops and when I stop, milk comes out. I have to come back to the hospital.” “Most of the difficulties people are dealing with here is that milk is not coming out at all. Which is something that is uncommon, and I didn’t hear about back home.” “My baby doesn’t want to take the breastfeeding. I do not know why.” “I didn’t want to stop during that time, but my child just stopped taking milk”
**Work-related breastfeeding barriers** “The reason I stopped was because of COVID. I was going to work and coming home and was scared of the virus.” “In my work, they don’t allow us to go pump, and I didn’t know that I have the right to request for that.” “The challenges have been work. Because I know back home, you are given 3 months. That is not the same thing here. I have to go back to work before the 3-month period. And that was really challenging.” “—it becomes a real challenge when after 2 months you have to go back to work—on top of taking care of everyone in the house, now you have to go back to work, and you come back tired. The baby has also missed the breast for so long and then there’s formula. The baby also doesn’t want to take the breast they go straight to the milk because they have been there all-day drinking formula.” “Going to the bathroom because the breast is hurting as we need to change the breast bandages. If you go more than 3 times you get in trouble. So, if you have to go and start pumping then you’ll get fired. So, we don’t even have room for that.” “It is also uncomfortable with me going back and forth to work and all that. So even though my breast keeps hurting I still go to work like that…. I feel like there’s a lot of women who are suffering.” “The inconvenience is after work I have so much milk in my breast. Even when I am trying to give it to the baby there is too much at once and the baby isn’t taking it very well. So, what I do right now is that when I come back from work, I will take the one that is already in the breast out and throw it away…… But I do not want to give them the milk from earlier in the day.” “With the type of work I do, I need to clean my breast before breastfeeding. I need to go home, take a shower, and then breastfeed.”
**Attitude towards the use of breast pumps** “Women in the community are not used to using the pump for our breast. It is scary, and we don’t know what will happen to the breast by using the pump. “When I pump and put the milk in the bottle and give my baby later, does it not give an infection and stuff to the babies?” “I am scared of the pump. It feels like we should let the baby breastfeed themselves. Also, I have a question. I want to know if we pump the breast, is it still going to have the same quality as if the child is taking milk from the breast?” ‘’I only use it once; I don’t like it because it hurts. But I was forced to use it when I had my baby because breast milk was not coming out and the nurse had to do something for the baby to get the milk and she used that.” “I can never use the pump because I feel like giving the child the breast milk connects them. And when they have a fever or anything like that you can easily know and feel it when you are giving breast milk” “I used the electrical pump or the manual one, but milk never comes out.” I do not like the idea of pumping the milk and leaving that for the child. Even for someone else to give it to the child. I like to breastfeed my child right away because when the milk comes out of my breast it is warm and has lots of vitamins compared to when it has already been pumped.” ”You can still pump the breast milk and leave it at home for your child. It’s as if you were present. That is one of the benefits. I can pump as much as I can, store it in the refrigerator. We know the time and everything. My son or my daughter will be able to get the breast milk as if I were home.” “I got help for my first kids. They even give me a pump. But I didn’t feel comfortable to use the pump, so I didn’t use it. They told me that the pump may help to get enough milk.” “Pumping is usually hard. Pumping the breast to give the child later. When I’m already pregnant and about to deliver the child, I already have an injury and a little pain around the nipple.”
**Attitude towards the use of donor milk** “I am against it. I would rather use formula instead of using another person’s breast milk.” “Even if the doctor says that milk is clean and free of bacteria, I still can’t give my child somebody else’s milk”.
**Lactation support and culturally sensitive hospital care** “Because when I came to America, I had two children already and nobody explained or gave me any education regarding breastfeeding.” “I have 3 kids, the first one breastfed for 12 months. The second was 11 months because of COVID… For the third one, I only breastfed for 3 weeks and stopped because the doctor asked me to stop breastfeeding because I had high blood glucose. The doctor decided that I should stop breastfeeding.” “It would be more important if the doctor during any consultation to share any information possible. To let the mother know that breastfeeding is important for her child, and it is beneficial for both of them to continue to do it.” “When I had my baby back home every time I went to the doctor, I received an advice session where they gave help and information on breastfeeding. The doctors explained to us why it was important to breastfeed and what breastfeeding can prevent and all those details. But here, the doctors ask more yes, or no questions and it is more about what you want to do. Do you want to breastfeed or give formula? And when they are meeting someone who wants to preserve shape then they just give formula like there is no issue with that.” “When I delivered back home, I knew what to do. But most people, when they deliver here, they do not know what to do because they are not educated. They only get the short visit when people come into your room. And when we go home, we just feel kind of lost. Because there is nowhere else we can go and WIC appointment are not always easily available.”
**Tailored community education needs** “The focus group like this will help people get educated on breastfeeding.“ “I feel like it would be best for you to contact every community’s president, you know the woman African community. They all have communities of women, and it would be best for us to go to them. And then reach out to the president of each community, they are more likely to relay the information to all the women in that community.” “There isn’t any website. Usually, friends just share it.” “I do agree with the usage of social media platforms. Like sharing videos on the social media platforms to reach a whole lot of people.” “Communication is key… In programs like WIC if they can communicate more with the new mother. To let them know the benefit of breastfeeding their child and the consequences if they do not do so. It would be better if there were translators available there for every language. For most of them they only have people who only speak English, and this doesn’t help people who don’t speak English as they won’t understand everything.”
